# Evaluating the Association of Eight Polymorphisms with Cancer Susceptibility in a Han Chinese Population

**DOI:** 10.1371/journal.pone.0132797

**Published:** 2015-07-15

**Authors:** Ying Dong, Jing Chen, Zhiqiang Chen, Chaoyong Tian, Huaisheng Lu, Jigang Ruan, Wenjun Yang

**Affiliations:** 1 Medical Oncology Department of General Hospital, Ningxia Medical University, Yinchuan, Ningxia, China; 2 Key Laboratory of Ningxia Reproduction and Heredity, Ningxia Medical University, Yinchuan, Ningxia, China; 3 Radiology Department of General Hospital, Ningxia Medical University, Yinchuan, Ningxia, China; 4 Pathology Department of Ningxia People’s Hospital, Yinchuan, Ningxia, China; 5 Digestive Department of General Hospital, Ningxia Medical University, Yinchuan, Ningxia, China; University of Cincinnati College of Medicine, UNITED STATES

## Abstract

**Background:**

The identification of susceptibility genes for specific types of cancer can provide necessary information for the complete characterization of cancer syndromes. Eight single nucleotide polymorphisms (SNPs), rs465498, rs17728461, rs4488809, rs753955, rs13361707, rs9841504, rs2274223, and rs13042395, were reported by genome wide association studies (GWASs) to be closely related to the susceptibility of lung cancer (LC), gastric cancer (GC) or esophageal cancer (EC) in Han population from northern or southern China. However, Chinese Han people from different geographic areas may have different genetic backgrounds. This study aims to assess the genetic associations of the eight SNPs mentioned above with three cancers risk in a Han population from northwest China.

**Methods:**

A total of 186 cancer-free controls and 436 cases with non-small cell lung cancer (NSCLC) (159 cases), non-cardia GC (167 cases) or EC (110 cases) were enrolled in this study. Chi-square test and polytomous logistic regression analyses were used to estimate the association between eight cancer-related SNPs and three cancers in a Han Chinese population from northwest China. The logistic regression results were adjusted for confounding factors and Benjamini and Hochberg False Discovery Rate (FDR) method was used to adjust the multiple hypothesis tests. Association analyses by cigarette smoking or alcohol drinking status were analyzed by crossover analyses.

**Results:**

One of the eight SNPs, rs17728461 was associated with NSCLC susceptibility (in a heterozygous model, OR = 0.44, 95% CI = 0.27–0.72, *p* = 0.001). Two SNPs, rs753955 and rs13042395, were associated with the risk of non-cardia GC in different genetic models (*p* < 0.05). No SNPs were associated with EC. The crossover analyses showed that the rs13042395 CT genotype, combined with cigarette smoking or alcohol drinking, could further increase the risk for non-cardia GC (*p* < 0.05).

**Conclusions:**

These results indicated that rs17728461 may be specifically associated with the risk of NSCLC. rs753955 and rs13042395 were specifically associated with susceptibility to non-cardia GC in Ningxia Han Chinese. Susceptibility-associated polymorphisms in the northwestern Han Chinese were not very consistent with those in the northern Han Chinese or southern Han Chinese. The validation of these findings with a functional evaluation and a larger population is still required.

## Introduction

Cancer is now understood to have both a genetic and an environmental component. Studies of hereditary cancer syndromes and targeted and genome-wide mutation analyses have provided ample evidence for the participation of genetic alterations in carcinogenesis [1]. Understanding genetic factors in relation to cancer is important because identifying such factors might be useful for risk prediction and for the development of chemo-preventive agents and other preventive measures [2].

Until now, genome-wide association studies (GWASs), a powerful method to investigate the genetic determinants of complex diseases, have successfully identified hundreds of SNPs related to the risk of cancers [3], including lung cancer (LC), gastric cancer (GC), and esophageal cancer (EC). The GWAS by Hu *et al* indicated that four SNPs, rs465498 at 5p15.33, rs17728461 at 22q12, rs4488809 at 3q28, and rs753955 at 13q12, were associated with LC risk in Han Chinese [4]. Two new SNPs, rs13361707 at 5q13.1 and rs9841504 at 3q13.31, identified by Shi *et al*, were significantly associated with the risk of GC [5]. Two SNPs, rs2274223 at 10q23 and rs13042395 at 20p13, were associated with risk of EC in a large number of Han Chinese [6]. However, the Han populations that were enrolled in these three GWASs were mainly selected from northern China (Beijing city) or southern China (Nanjing city of Jiangsu province). Chinese Han people from different places may have different genetic backgrounds due to their complex origins and long history of interaction with many surrounding ethnic groups [7]. Therefore, the Han people from the Ningxia Hui autonomous region, which is located in northwest China, may have a different genetic background from those in northern or southern China. To further explore the associations and specificities of the eight SNPs (rs465498, rs17728461, rs4488809, rs753955, rs13361707, rs9841504, rs2274223, and rs13042395) mentioned above with the susceptibility of three cancers in the Han Chinese population in the Ningxia region of northwest China, a hospital-based case-control study was performed.

## Samples and methods

### Study population and data collection

All of the samples were from Ningxia Han residents whose ancestral native living places were Ningxia Hui Autonomous Region and at least three generations of their families were Han people. Patients with primary non-small cell lung cancer (NSCLC), non-cardia GC or EC were recruited between 2009 and 2012 in the General Hospital of Ningxia Medical University (Ningxia Region, China). All of the enrolled cancer patients were diagnosed by pathological means, and their diagnoses were histologically confirmed. Additionally, patients with chronic diseases, conditions that involved vital organs, or severe endocrinological, metabolic, or nutritional diseases were excluded from this study. Patients were also excluded if they had received any blood transfusion during the past 6 months, immunosuppressive therapy, chemotherapy or radiation therapy.

Healthy, unrelated individuals were randomly recruited as control subjects from the health check-up center at the General Hospital during the same period. The inclusion criterion for the controls was the absence of cancer history. None of these healthy people had a history of contact with a strong carcinogen like asbestos, arsenic trioxide, benzene and so on.

Each subject was personally questioned by trained interviewers using a pre-tested questionnaire to obtain information on demographic data, including the age at diagnosis, gender, ethnicity, family history of cancer, residential region, occupation, living and eating habits (*e*.*g*., cigarette smoking and alcohol consumption). Individuals who smoked one cigarette per day for more than 1 year were classified as smokers, and those who had three times or more alcoholic drinks a week for more than 6 months were defined as alcohol drinkers. After the interview, 2-ml samples of venous blood were collected from each participant for DNA preparation and genotyping. Finally, 440 cases (including 162 NSCLC patients, 168 non-cardia GC patients, and 110 EC patients) and 186 cancer-free controls were included in this study.

This hospital-based case-control study was approved by the Medical Ethics Review Committee of Ningxia Medical University (Ningxia Region, China). Signed informed consent was obtained from each participant.

### SNP selection and genotyping

Literature on GWASs published until December 2012 was reviewed, and candidate SNPs that were previously reported to be associated with LC (rs465498, rs753955, rs17728461, and rs4488809), GC (rs13361707 and rs9841504) and EC (rs2274223 and rs13042395) were selected for investigation. Genomic DNA was extracted from the peripheral blood leukocytes of the participants using a QIAamp DNA Mini kit (Qiagen, Hilden, Germany) following the manufacturer’s instructions and stored at -20°C until use.

The SNP genotyping work was performed by an improved multiplex ligase detection reaction method (iMLDR, Genesky Bio-Tech Cod., Ltd., Shanghai, China) as previously described [8]. The primers for the polymerase chain reaction (PCR) and the probes for the LDR were listed in Table 1. Two negative controls were set: one with double-distilled water as template and the other with DNA sample without primers while keeping all other conditions the same in one plate. Duplicate tests were designed and the results were consistent. The call rates in the genotyping of all these eight SNPs were above 99% (Table 2). Three NSCLC cases and one non-cardia GC cases were excluded due to low genotyping quality. rs9841504 and rs2274223 were also selected and genotyped by both iMLDR method and direct sequencing method to evaluate concordance of the genotyping. The concordance rates in the genotyping of two SNPs were 100%. Finally, 436 cases (159 NSCLC patients, 167 non-cardia GC patients, and 110 EC patients) and 186 cancer-free controls were included in this study.

**Table 1:** The primers for polymerase chain reaction (PCR) and probers for LDR.

**SNPs**	**Primers or probes**	**Sequences**
rs465498	Upstream primer	GGGAATCCTCAGGCGAAGAGAG
	Downstream primer	CCCCTCAGCTGTGGAAAGTGAA
	FG	TCTCTCGGGTCAATTCGTCCTT GCAGATGGCCATCTTACTCTTTTCG
	FA	TGTTCGTGGGCCGGATTAGT GCAGATGGCCATCTTACTCTTTCCA
	FP	CAGGGGGCAAAATCACACTCT TTTTTTTTTT
rs17728461	Upstream primer	GGTGCCAAGTGGGGAAGGTAGA
	Downstream primer	TGGTCTGATAACCACCCGAATCA
	RC	TCTCTCGGGTCAATTCGTCCTT GAAGCAGGAGAGTGGAATCTGAGACATG
	RG	TGTTCGTGGGCCGGATTAGT GAAGCAGGAGAGTGGAATCTGAGACATC
	RP	TGCAGATAAAACTTCTCCACTGAGG TTTTTTTTTTTT
rs4488809	Upstream primer	GCAGGCCACATGCAAAAAAAGTC
	Downstream primer	AGGAGGAACAGCCGCTGAGAGT
	FC	TCTCTCGGGTCAATTCGTCCTT CAASCATCTGCTCTTGAGGCAGTACAC
	FT	TGTTCGTGGGCCGGATTAGT CAASCATCTGCTCTTGAGGCAGTACAT
	FP	TGAAACAGTGATTCTTAYTACTAGTTTCTGTTAAAGT TTTTTTTTTT
rs753955	Upstream primer	TTTTGCCCGTACCTCTCCTTGT
	Downstream primer	TCATGGCTTTTTTCCCAAAGGT
	FG	TCTCTCGGGTCAATTCGTCCTT tgcttgggaaggttggtcttgttgtG
	FA	TGTTCGTGGGCCGGATTAGT tgcttgggaaggttggtcttgttgtA
	FP	ttcaagccttcacatgattggac TTTTTTTTTTTTT
rs13361707	Upstream primer	TTCAAGCACACCAAGGGCATTC
	Downstream primer	GGCAGGGGCAAATCTGTATCTT
	FC	TTCCGCGTTCGGACTGATAT GCATTCcaaatacccatgagcctcC
	FT	TACGGTTATTCGGGCTCCTGT GCATTCcaaatacccatgagcctcT
	FP	atcagcttaagcaataaaacattacaaatacaa TTTTTTTTTT
rs9841504	Upstream primer	TTTTGCCCGTACCTCTCCTTGT
	Downstream primer	TCATGGCTTTTTTCCCAAAGGT
	RC	TCTCTCGGGTCAATTCGTCCTT TGTTAAGGAGCAGTTGAAAGGATTACAGG
	RG	TGTTCGTGGGCCGGATTAGT TGTTAAGGAGCAGTTGAAAGGATTACAGC
	RP	TAAAAATATATAGGGAAAAAGGGAAGAAWGTTACA TTTTTTT
rs2274223	Upstream primer	CACAAAGCCCATCCATCGAAAC
	Downstream primer	AGCAAGCATGGGTGAGGCTGTA
	RG	TTCCGCGTTCGGACTGATAT CGAAGAAATACAAGATCTTCGAAGTGAAAGC
	RA	TACGGTTATTCGGGCTCCTGT CGAAGAAATACAAGATCTTCGAAGTGAAGGT
	RP	GRAACAGAAACTGCTCGTTCCA TTTTTTTTTTTT
rs13042395	Upstream primer	CCCAGGGAAAGACAATGCTGTG
	Downstream primer	GGAGCATCACAGTCCCATGTTCA
	FC	TTCCGCGTTCGGACTGATAT ACCAGGGCCAGTGCACCTTC
	FT	TACGGTTATTCGGGCTCCTGT ACCAGGGCCAGTGCACCCTT
	FP	ATTGTGTGGGCTGGGCCATC TTTTTTTTTTTTT

**Table 2:** Candidate SNPs in this study.

**SNP No.**	**Loc.**	**Gene**	**Category**	**ref**	**Call Rate (%)**	**MAF in control group**			**GENO**	**HWE *p***
						**A1**	**GWAS**	**our**		
rs465498	5p15.33	*CLPTM1L*	intron	4	99.60	G	0.16	0.19	9/54/123	0.35
rs17728461	22q12	*HORMAD2-LIF*	intergenic	4	99.49	G	0.17	0.26	9/79/98	0.19
rs4488809	3q28	*TP63*	intron	4	99.29	C	0.47	0.48	41/97/48	0.66
rs753955	13q22	*MIPEP-TNFRSF19*	intergenic	4	99.49	G	0.28	0.30	13/85/88	0.29
rs13361707	5q13.1	*PRKAA1*	intron	5	99.19	C	0.48	0.47	41/91/54	0.88
rs9841504	3q13.31	*ZBTB20*	intron	5	99.19	G	0.14	0.13	4/40/142	0.51
rs2274223	10q23	*PLCE1*	nonsynonymous	6	99.39	G	0.20	0.23	7/73/106	0.23
rs13042395	20p13	*C20orf54*	intron	6	99.39	T	0.28	0.24	15/59/112	0.10

MAF: Minor allele frequency; HWE: Hardy-Weinberg equilibrium; α = 0.05.

### Statistical analysis

A chi-squared test was used to evaluate the differences in the distributions of demographic characteristics, selected variables and genotypes between the cases and controls. Continuous variables were analyzed using Student’s *t* test. Genotype frequencies in the control subjects for each SNP were tested for departure from Hardy-Weinberg equilibrium (HWE) using the goodness-of-fit *χ^2^* test. Polytomous logistic regression analyses were used to estimate the adjusted odds ratios (ORs) and the 95% confidence intervals (CIs) for the association between genetic variants and cancer risk, as evaluated by different genetic models (additive, heterozygous, homozygous, and dominant and recessive) adjusted for age, gender, family history of cancer, cigarette smoking and alcohol drinking status. Benjamini and Hochberg False Discovery Rate (FDR) method was used to adjust the multiple hypothesis tests. Crossover analyses were used to conduct association analyses between rs13042394 and non-cardia GC by cigarette smoking or alcohol drinking status. All of the statistical analyses were performed using Microsoft Excel 2007, SPSS 17.0 statistical package (SPSS, Chicago, IL) and the plink-1.07-dos program. The standard α = 0.05.

## Results

### Demographic data of the participants

Demographic data including age, gender, and family history of cancer, cigarette smoking, and alcohol drinking for all subjects recruited in this study were summarized in Table 3. There were significant differences between the NSCLC, non-cardia GC or EC group and control group in age (*p* < 0.05). No statistical difference was found in gender between the NSCLC group (males account for 64.2%) and the control group (males account for 60.8%). However, the percentages of males in non-cardia GC (72.5%) and EC (74.5%) were higher than that in the control group (60.8%). The difference between NSCLC and control group in alcohol drinking was not significant but more NSCLC patients were cigarette smokers (*p* = 0.027). Statistically significant differences were found between non-cardia GC or EC and cigarette smoking, alcohol drinking or family history of cancer (*p* < 0.05).

**Table 3:** Demographic data of all subjects recruited in this study.

**Total**	**Age**		**Gender**		**Cigarette Smoking**		**Acohol Drinking**		**Family History of cancer**	
	**Mean ± SD**	***p*** ^*a*^	**Male (%)**	***p*** ^*b*^	**YES (%)**	***p*** ^*b*^	**YES (%)**	***p*** ^*b*^	**YES (%)**	***p*** ^*b*^
Control (n = 186)	50.99 ± 16.41		113 (60.8)		57 (30.6)		27 (14.5)		0 (0.0)	
NSCLC (n = 159)	59.04 ± 10.45	**< 0.001**	102 (64.2)	0.516	67 (42.1)	**0.027**	27 (17.0)	0.530	0 (0.0)	-
GC (n = 167)	57.38 ± 10.69	**< 0.001**	121 (72.5)	**0.020**	94 (56.3)	**< 0.001**	59 (35.3)	**< 0.001**	6 (3.6)	**0.009**
EC (n = 110)	62.05 ± 9.10	**< 0.001**	82 (74.5)	**0.016**	60 (54.5)	**< 0.001**	38 (34.5)	**< 0.001**	4 (3.6)	**0.009**

NSCLC, non-small cell lung cancer;GC, gastric cancer; EC, esophageal cancer.

^a^ p value of t-test

^b^ p value of χ2 test

α = 0.05.

The results of the logistic regression analyses in Table 4 indicated that the association between NSCLC and cigarette smoking exposure was significant(OR = 1.65, 95% CI = 1.06–2.57, *p* = 0.027). Similar results were found between non-cardia GC or EC and cigarette smoking exposure (OR = 2.91, 95% CI = 1.88–4.51, *p* < 0.001, and OR = 2.72, 95% CI = 167–4.42, *p* < 0.001, respectively) or alcohol drinking exposure (OR = 3.22, 95% CI = 1.92–5.39, *p* < 0.001, and OR = 3.11, 95% CI = 1.76–5.48, *p* < 0.001, respectively).

**Table 4:** Association between demographic data and cancer.

	**NSCLC**		**non-cardia GC**		**EC**	
	**OR (95%CI)**	***p*** ^*a*^	**OR (95%CI)**	***p*** ^*a*^	**OR (95%CI)**	***p*** ^*a*^
Age	1.04 (1.03–1.06)	**<0.001**	1.03 (1.02–1.05)	**<0.001**	1.06 (1.04–1.08)	**< 0.001**
Gender ^b^	1.16 (0.75–1.79)	0.516	1.70 (1.08–2.66)	**0.021**	1.89 (1.12–3.18)	**0.016**
Cigarette smoking	1.65 (1.06–2.57)	**0.027**	2.91 (1.88–4.51)	**<0.001**	2.72 (1.67–4.42)	**< 0.001**
Alcohol drinking	1.21 (0.67–2.15)	0.530	3.22 (1.92–5.39)	**<0.001**	3.11 (1.76–5.48)	**< 0.001**

NSCLC, non-small cell lung cancer; non-cardia GC, non-cardia gastric cancer; EC, esophageal cancer.

^a^ p values of unconditional logistic regression analysis, α = 0.05

^b^ female as reference.

### Genetic distribution of the SNPs in a case-control study

Basic information of the selected SNPs was shown in Table 2. All of the SNPs had distributions within the parameters of HWE for the control population. The genotype frequencies of the eight SNPs in the cases and controls were shown in Table 5.

**Table 5:** The genotype frequencies of the eight SNPs.

**Variables**	**Controls (n = 186)**	**NSCLC (n = 159)**		**non-cardiac GC (n = 167)**		**EC (n = 110)**	
	**n (%)**	**n (%)**	***p***	**n (%)**	***p***	**n (%)**	***p***
GWAS rs465498			0.382*		**0.050** *		0.155*
AA	123 (66.1)	113 (71.1)	ref.	123 (73.7)	ref.	82 (74.5)	ref.
GA	54 (29.0)	39 (24.5)	0.330	42 (25.1)	0.299	24 (21.8)	0.152
GG	9 (4.8)	7 (4.4)	0.749	2 (1.2)	**0.039**	4 (3.6)	0.509
GWAS rs17728461			0.269*		0.661*		0.152*
CC	98 (52.7)	104 (65.4)	ref.	92 (55.1)	ref.	66 (60.0)	ref.
CG	79 (42.5)	39 (24.5)	**0.001**	66 (39.5)	0.598	40 (36.4)	0.255
GG	9 (4.8)	16 (10.1)	0.237	8 (4.8)	0.914	3 (2.7)	0.296
GWAS rs4488809			0.933*		0.788*		0.195*
CC	41 (22.0)	38 (23.9)	ref.	37 (22.2)	ref.	34 (30.9)	ref.
CT	97 (52.2)	88 (55.3)	0.603	90 (53.9)	0.679	48 (43.6)	0.764
TT	48 (25.8)	33 (20.8)	0.959	40 (24.0)	0.798	26 (23.6)	0.204
GWAS rs753955			0.705*		**0.001** *		0.244*
AA	88 (47.3)	75 (47.2)	ref.	59 (35.3)	ref.	49 (44.5)	ref.
GA	85 (45.7)	69 (43.4)	0.829	74 (44.3)	0.259	45 (40.9)	0.844
GG	13 (7.0)	15 (9.4)	0.459	34 (20.4)	**0.000**	15 (13.6)	0.078
GWAS rs13361707			0.626*		**0.005** *		0.786*
TT	41 (22.0)	46 (28.9)	ref.	62 (37.1)	ref.	23 (20.9)	ref.
CT	91 (48.9)	71 (44.7)	0.731	68 (40.7)	0.745	51 (46.4)	0.759
CC	54 (29.0)	41 (25.8)	0.591	37 (22.2)	**0.007**	33 (30.0)	0.802
GWAS rs9841504			0.953*		0.390*		0.612*
CC	142 (76.3)	119 (74.8)	ref.	133 (79.6)	ref.	85 (77.3)	ref.
CG	40 (21.5)	35 (22.0)	0.869	32 (19.2)	0.553	23 (20.9)	0.892
GG	4 (2.2)	3 (1.9)	0.886	2 (1.2)	0.466	1 (0.9)	0.424
GWAS rs2274223			0.143*		0.213*		0.793*
AA	106 (57.0)	106 (66.7)	ref.	93 (55.7)	ref.	65 (59.1)	ref.
GA	73 (39.2)	46 (28.9)	**0.047**	56 (33.5)	0.555	39 (35.5)	0.586
GG	7 (3.8)	7 (4.4)	1.000	18 (10.8)	**0.017**	5 (4.5)	0.801
GWAS rs13042395			0.070*		**0.016** *		0.792*
CC	112 (60.2)	76 (43.1)	ref.	72 (43.1)	ref.	64 (58.2)	ref.
CT	59 (31.7)	70 (44.0)	**0.015**	83 (49.7)	**0.001**	40 (36.4)	0.507
TT	15 (8.1)	13 (8.2)	0.547	12 (7.2)	0.598	5 (4.5)	0.313

NSCLC, non-small cell lung cancer; non-cardia GC, non-cardia gastric cancer; EC, esophageal cancer.

* p values for Cochran-Armitage trend test, α = 0.05.

p values for prtitions of chi-square test, α = 0.0125.

### Association between SNPs and cancers

Polytomous logistic regression analyses were used to estimate the association between SNPs and cancer risk using different genetic models. The results were listed in Tables 6 and 7, and all of the results were adjusted by age, gender, family history of cancer, cigarette smoking, and alcohol drinking.

**Table 6:** Association between SNPs and cancer.

			**LC rs465498 A>G**		**LC rs17728461 C>G**		**LC rs4488809 T>C**		**LC rs753955 A>G**	
			**OR (95%CI)**	***p*** ^*a*^	**OR (95%CI)**	***p*** ^*a*^	**OR (95%CI)**	***p*** ^*a*^	**OR (95%CI)**	***p*** ^*a*^
**NSCLC**	Additive		0.79 (0.53–1.19)	0.262	0.88 (0.56–1.17)	0.258	0.97 (0.71–1.32)	0.846	1.10 (0.79–1.54)	0.586
		AA	1		1		1		1	
	Heterozygote	AB	0.75 (0.45–1.24)	0.256	**0.44 (0.27–0.72)**	**0.0010**	1.11 (0.64–1.90)	0.718	0.94 (0.59–1.49)	0.788
	Homozygote	BB	0.784 (0.28–2.23)	0.648	1.91 (0.75–4.86)	0.484	0.92 (0.48–1.77)	0.800	1.52 (0.66–3.46)	0.324
	Dominant	AB+BB	0.75 (0.47–1.21)	0.243	0.57 (0.36–0.90)	0.015	1.04 (0.62–1.62)	0.896	1.02 (0.66–1.58)	0.942
		AA+AB	1		1		1		1	
	Recessive	BB	0.84 (0.30–2.37)	0.741	2.45 (1.00–6.02)	0.050	0.88 (0.52–1.51)	0.642	1.54 (0.69–3.41)	0.290
**non-cardia GC**	Additive		0.66 (0.43–1.01)	0.054	0.95 (0.67–1.36)	0.783	1.02 (0.75–1.40)	0.892	**1.86 (1.34–2.58)**	**0.0002**
		AA	1		1		1		1	
	Heterozygote	AB	0.79 (0.48–1.30)	0.342	0.91 (0.58–1.44)	0.691	0.93 (0.54–1.59)	0.780	1.30 (0.81–2.10)	0.277
	Homozygote	BB	0.21 (0.04–1.03)	0.054	1.04 (0.36–3.01)	0.937	1.02 (0.54–1.94)	0.948	**4.69 (2.18–10.08)**	**0.0001**
	Dominant	AB+BB	0.70 (0.43–1.13)	0.144	0.92 (0.59–1.43)	0.695	0.97 (0.58–1.62)	0.913	1.72 (1.09–2.70)	0.019
		AA+AB	1		1		1		1	
	Recessive	BB	0.23 (0.05–1.09)	0.064	1.07 (0.39–2.99)	0.892	1.09 (0.64–1.86)	0.745	**4.04 (1.99–8.23)**	**0.0001**
**EC**	Additive		0.73 (0.45–1.18)	0.198	0.79 (0.52–1.22)	0.295	1.22 (0.85–1.74)	0.285	1.37 (0.94–2.00)	0.105
		AA	1		1		1		1	
	Heterozygote	AB	0.70 (0.39–1.27)	0.246	0.77 (0.45–1.31)	0.334	0.80 (0.42–1.53)	0.508	0.99 (0.58–1.70)	0.964
	Homozygote	BB	0.64 (0.18–2.35)	0.484	0.62 (0.15–2.56)	0.507	1.40 (0.69–2.83)	0.350	2.59 (1.08–6.20)	0.033
	Dominant	AB+BB	0.70 (0.40–1.22)	0.204	0.76 (0.45–1.26)	0.286	1.00 (0.55–1.82)	0.989	1.20 (0.72–2.00)	0.477
		AA+AB	1		1		1		1	
	Recessive	BB	0.71 (0.20–2.51)	0.597	0.67 (0.17–2.67)	0.568	1.66 (0.93–2.97)	0.085	2.65 (1.15–6.08)	0.022

NSCLC, non-small cell lung cancer; non-cardia GC, non-cardia gastric cancer; EC, esophageal cancer.

Benjamini and Hochberg False Discovery Rate (FDR) method was used to adjust the multiple hypothesis tests, standard α = 0.05.

^a^ adjusted by age, gender, family history of cancer(non-cardia GC and EC), cigarette smoking, and alcohol drinking.

**Table 7:** Association between SNPs and cancer.

			**GC rs13361707 T>C**		**GC rs9841504 C>G**		**EC rs2274223 A>G**		**EC rs13042395 C>T**	
			**OR (95%CI)**	***p*** ^*a*^	**OR (95%CI)**	***p*** ^*a*^	**OR (95%CI)**	***p*** ^*a*^	**OR (95%CI)**	***p*** ^*a*^
**NSCLC**	Additive		1.04 (0.76–1.42)	0.809	1.05 (0.66–1.67)	0.837	0.69 (0.47–1.01)	0.055	1.37 (0.96–1.94)	0.079
		AA	1		1		1		1	
	Heterozygote	AB	0.96 (0.57–1.61)	0.865	1.07 (0.63–1.82)	0.804	1.07 (0.63–1.82)	0.804	1.79 (1.12–2.88)	0.016
	Homozygote	BB	1.06 (0.57–1.96)	0.860	1.04 (0.21–5.10)	0.962	0.70 (0.23–2.16)	0.533	1.21 (0.53–2.76)	0.656
	Dominant	AB+BB	0.99 (0.61–1.61)	0.978	1.06 (0.63–1.78)	0.818	0.61 (0.38–0.96)	0.032	1.67 (1.07–2.61)	0.023
		AA+AB	1		1		1		1	
	Recessive	BB	1.12 (0.67–1.88)	0.656	1.01 (0.21–4.85)	0.993	0.87 (0.29–2.61)	0.804	0.96 (0.43–2.15)	0.922
**non-cardia GC**	Additive		1.40 (1.03–1.92)	0.033	0.86 (0.53–1.39)	0.533	1.20 (0.84–1.72)	0.309	1.58 (1.11–2.23)	0.010
		AA	1		1		1		1	
	Heterozygote	AB	1.10 (0.64–1.91)	0.734	0.89 (0.52–1.53)	0.673	0.89 (0.52–1.53)	0.673	**2.54 (1.57–4.10)**	**0.0001**
	Homozygote	BB	1.78 (0.96–3.31)	0.068	0.62 (0.10–3.69)	0.598	2.23 (0.85–5.81)	0.102	1.21 (0.51–2.84)	0.666
	Dominant	AB+BB	1.32 (0.80–2.21)	0.275	0.86 (0.51–1.47)	0.592	1.06 (0.68–1.66)	0.800	**2.25 (1.43–3.53)**	**0.0004**
		AA+AB	1		1		1		1	
	Recessive	BB	1.75 (1.07–2.87)	0.026	0.64 (0.11–3.82)	0.622	2.39 (0.94–6.10)	0.068	0.82 (0.36–1.88)	0.636
**EC**	Additive		0.84 (0.58–1.20)	0.340	0.94 (0.55–1.64)	0.837	0.87 (0.57–1.34)	0.541	0.99 (0.65–1.51)	0.977
		AA	1		1		1		1	
	Heterozygote	AB	0.92 (0.51–1.67)	0.779	1.01 (0.55–1.88)	0.971	1.01 (0.55–1.88)	0.971	1.37 (0.80–2.37)	0.254
	Homozygote	BB	0.69 (0.33–1.43)	0.314	0.56 (0.6–5.63)	0.624	0.81 (0.23–2.84)	0.743	0.54 (0.18–1.66)	0.283
	Dominant	AB+BB	0.84 (0.48–1.47)	0.550	0.98 (0.53–1.79)	0.938	0.86 (0.51–1.44)	0.558	1.19 (0.71–1.99)	0.509
		AA+AB	1		1		1		1	
	Recessive	BB	0.76 (0.41–1.40)	0.377	0.56 (0.06–5.54)	0.618	0.87 (0.26–2.96)	0.827	0.49 (0.16–1.46)	0.200

NSCLC, non-small cell lung cancer; non-cardia GC, non-cardia gastric cancer; EC, esophageal cancer.

Benjamini and Hochberg False Discovery Rate (FDR) method was used to adjust the multiple hypothesis tests, standard α = 0.05.

^a^ adjusted by age, gender, family history of cancer(non-cardia GC and EC), cigarette smoking, and alcohol drinking.

After adjusted by Benjamini and Hochberg False Discovery Rate (FDR) method, only one of the eight SNPs, rs17728461 in the *HORMAD2*-*LIF* gene was associated with NSCLC risk. The CG heterozygote of rs17728461, when compared to the CC homozygote, was associated with a decreased risk of NSCLC (adjusted OR = 0.44, 95% CI = 0.27–0.72, *p* = 0.0010).

Two of the eight SNPs, rs753955 in the *MIPEP-TNFRSF19* gene and rs13042395 in the *C20orf54* gene, were associated with the susceptibility of non-cardia GC. rs753955 G-allele was associated with an increased risk of non-cardia GC (additive model: OR = 1.86, 95% CI = 1.34–2.58, *p* = 0.0002). In homozygote model, the non-cardia GC risk of rs753955 GG genotype carriers was 4.69 times than those who carry rs753955 AA genotype (OR = 4.69, 95% CI = 2.18–10.08, *p* = 0.0001) Same result was found in recessive model (OR = 4.04, 95% CI = 1.99–8.23, *p* = 0.0001). Similarly, rs13042395 CT genotype or CT + TT genotype were associated with an increased risk of non-cardia GC when compared with CC genotype (heterozygote model, OR = 2.54, 95% CI = 1.57–4.10, *p* = 0.0001; dominant model: OR = 2.25, 95% CI = 1.43–3.53, *p* = 0.0004).

However, no association between the eight SNPs and EC risk was observed in this study.

### Crossover analysis by cigarette smoking or alcohol drinking status

The results of crossover analyses by cigarette smoking or alcohol drinking status were illustrated in Table 8. Crossover analysis indicated that there was significantly increased risk of non-cardia GC among participants with rs13042395 CT+TT genotype who were also cigarette smokers or alcohol drinkers when compared to those non-cigarette smokers or non-alcohol drinkers who carried the rs13042395 CC genotype (adjusted OR = 6.03, 95% CI = 2.63–13.86, *p* < 0.0001, and adjusted OR = 4.62, 95% CI = 1.87–11.39, *p* = 0.0009, respectively). When focused on rs13042395 CT carriers who were also cigarette smokers or alcohol drinkers, the risk of non-cardia GC further increased (adjusted OR = 6.75, 95% CI = 2.78–16.36, *p* < 0.0001, and adjusted OR = 7.47, 95% CI = 2.50–22.32, *p* = 0.0003, separately).

**Table 8:** Crossover analysis by cigarette smoking or alcohol drinking status.

**Smoking/Drinking**	**Genotype**	**case (n = 167)**	**Control (n = 186)**	**Crude OR (95% CI)**	***p***	**Adjusted OR** ^a^ **(95% CI)**	***p*** ^a^
Cigarette Smoking		n (%)	n (%)				
(—)	CC	29 (17.4)	74 (39.8)	1		1	
(—)	TT+CT	44 (26.3)	55 (29.5)	2.04 (1.14–3.66)	0.017	2.32 (1.25–4.32)	0.008
(+)	CC	43 (25.7)	38 (20.4)	2.89 (1.57–5.33)	**0.0007**	2.72 (1.24–6.00)	0.013
(+)	TT+CT	51 (30.5)	19 (10.2)	6.85 (3.47–13.52)	**< 0.0001**	6.03 (2.63–13.86)	**< 0.0001**
(—)	CC	29 (17.4)	74 (39.8)	1		1	
(—)	CT	38 (22.8)	45 (24.2)	2.15 (1.17–3.96)	0.013	2.58 (1.35–4.95)	0.004
(+)	CC	43 (25.7)	38 (20.4)	2.89 (1.56–5.33)	**0.0007**	2.52 (1.12–5.65)	**0.025**
(+)	CT	45 (26.9)	14 (7.5)	8.20 (3.92–17.15)	**< 0.0001**	6.75 (2.78–16.36)	**< 0.0001**
Acohol Drinking							
(—)	CC	45 (26.9)	94 (50.5)	1		1	
(—)	TT+CT	63 (37.7)	65 (34.9)	2.03 (1.23–3.33)	0.005	2.27 (1.34–3.87)	0.002
(+)	CC	27 (16.2)	18 (9.7)	3.13 (1.57–6.28)	0.001	2.03 (0.91–4.53)	0.084
(+)	TT+CT	32 (19.2)	9 (4.9)	7.43 (3.27–16.87)	**< 0.0001**	4.62 (1.87–11.39)	**0.0009**
(—)	CC	45 (26.9)	94 (50.5)	1		1	
(—)	CT	55 (32.9)	54 (29.0)	2.13 (1.27–3.57)	0.004	2.43 (1.40–4.23)	0.002
(+)	CC	27 (16.2)	18 (9.7)	3.13 (1.57–6.23)	0.001	2.09 (0.93–4.72)	0.075
(+)	CT	28 (16.8)	5 (2.7)	11.70 (4.24–32.30)	**< 0.0001**	7.47 (2.50–22.32)	**0.0003**

^a^ adjusted for sex, age, smoking, alcohol drinking and family history of cancer

α = 0.05.

## Discussion

Cancer is the second-leading cause of death and disability in the world, behind only heart disease [9]. According to the new version of International Agency Research on Cancer (IARC)’s online database GLOBOCAN 2012, among the more than two dozen distinct cancers that have been examined, LC, non-cardia GC, and EC rank in the top ten in incidence and mortality rates [10]. Approximately 40% of new cases and deaths from these three cancers in the world have occurred in China which makes LC, non-cardia GC and EC a great threat to human health in China [10].

To assess the associations between SNPs and cancers, a large number of studies have been conducted within the last decade in different ethnic populations, including the Han Chinese. The Han Chinese population is the largest ethnic group in China, composing 98% of the entire Chinese population [7]. Some studies have reported that the genetic background of the Han population in northern China is different from that in southern China [11–14]. Therefore, the Han Chinese population, a seemingly homogeneous population, actually has a complicated substructure. In this study, we analyzed the associations between the eight SNPs that were previously reported in GWASs from two Han populations in different geographical places and NSCLC, non-cardia GC and EC in a Han Chinese population from northwest China.

Among the eight SNPs, only rs17728461 was specifically associated with NSCLC in our study. rs17728461 is located at 22q12.2, approximately 38 kb downstream of the *LIF* gene that encodes leukemia inhibitory factor (LIF). LIF plays an important role in the process of LC through the signal transducer and activator of transcription 3 (STAT3) pathway [15, 16]. Hu *et al*’s GWAS first identified rs17728461 G-allele as a risk factor of LC in Han populations from northern and southern China. In contrast, however, our work found that rs17728461 G-allele was associated with a decreased risk of NSCLC in a Han population from northwest China. The allele information indicates that the minor allele frequency (MAF) of rs17728461 in the population from northwest China and the populations from northern and southern China varied widely (0.26 *vs*. 0.17).

Two other SNPs, rs753955 and rs13042395 were observed to be specifically and separately associated with non-cardia GC risk in this study. rs753955 is located in the *MIPEP* gene at 13q12.12, encoding mitochondrial intermediate peptidase (MIPEP). MIPEP may contribute to frataxin deficiency and iron utilization and the exact mechanism of MIPEP in carcinogenesis still needs to be addressed in the future [17]. Hu *et al*’s GWAS first reported that rs753955 G-allele was correlated with the susceptibility of LC in populations from both northern China and southern China. In our study, however, rs753955 G-allele was found to be related to non-cardia GC risk instead of LC risk in the Han population from northwest China. In addition, we also noticed that rs13042395 T-allele was associated with the increased risk of non-cardia GC. The results of crossover analysis by cigarette smoking or alcohol drinking status further indicated that rs13042395 CT carriers showed a significantly increased risk of non-cardia GC when they were also cigarette smokers or alcohol drinkers. rs13042395, located in the *C20orf54* gene at 20p13, was first reported as a risk factor for esophageal squamous cell carcinoma (ESCC) in Wang *et al*’s GWAS of a northern Han population. However, five studies from different groups, including ours, failed to find any association with EC [18–21]. Additionally, in a later study, Wang *et al* reported a correction that the published association for rs13042395 T-allele could not be replicated in additional analyses of data from the same region; the original finding might be the result of an inadequate control for population stratification using genetically unmatched subjects or, less likely, could have been due to chance alone [6].

Overall, we analyzed the associations between the reported eight SNPs and the risk of three cancers (NSCLC, non-cardia GC and EC) in a Han population from northwest China. Our statistical data showed that rs17728461 was specifically associated with the risk of NSCLC, rs753955 and rs13042395 were specifically related with susceptibility to non-cardia GC. The susceptibility polymorphisms in the northwestern Han Chinese were not very consistent with those in the northern or southern Han Chinese.

## Conclusions

In conclusion, our work and that of others have shown that susceptibility polymorphisms identified in GWASs can vary in different ethnic populations, even in very close Han populations living in different geographic areas. However, several limitations of our work should be mentioned. First, since all of the participants were enrolled from a hospital and the average age of the controls was also younger than those of the cases, therefore, selection bias cannot be excluded. Second, our results were obtained with a limited sample size, allowing us to draw only preliminary conclusions. Finally, functional assays are required for further studies. Therefore, the validation of these findings by functional evaluation and with a larger population is required.
